# A formal model accounting for measurement reliability shows attenuated effect of higher education on intelligence in longitudinal data

**DOI:** 10.1098/rsos.230513

**Published:** 2024-05-08

**Authors:** Kimmo Eriksson, Kimmo Sorjonen, Daniel Falkstedt, Bo Melin, Gustav Nilsonne

**Affiliations:** ^1^ School of Education, Culture and Communication, Mälardalen University, Västerås, Sweden; ^2^ Institute for Futures Studies, Stockholm, Sweden; ^3^ Department of Clinical Neuroscience, Karolinska Institutet, Stockholm, Sweden; ^4^ Institute of Environmental Medicine, Karolinska Institutet, Stockholm, Sweden; ^5^ Department of Psychology, Stockholm University, Stockholm, Sweden

**Keywords:** education, intelligence, reliability, simulation, mathematical model, ceiling effect

## Abstract

The effect of higher education on intelligence has been examined using longitudinal data. Typically, these studies reveal a positive effect, approximately 1 IQ point per year of higher education, particularly when pre-education intelligence is considered as a covariate in the analyses. However, such covariate adjustment is known to yield positively biased results if the covariate has measurement errors and is correlated with the predictor. Simultaneously, a negative bias may emerge if the intelligence measure after higher education has non-classical measurement errors as in data from the 1970 British Cohort Study that were used in a previous study of the effect of higher education. In response, we have devised an estimation method that used iterated simulations to account for both classical measurement errors in the covariate and non-classical errors in the dependent variable. Upon applying this method in a reanalysis of the data from the 1970 British Cohort Study, we find that the estimated effect of higher education diminishes to 0.4 IQ points per year. Additionally, our findings suggest that the impact of higher education is somewhat more pronounced in the initial 2 years of higher education, aligning with the notion of diminishing marginal cognitive benefits.

## 1. Introduction

Many studies have reported a positive effect of education on intelligence. A recent meta-analysis [1] described three research designs, which examine the effect of education on intelligence at different educational stages: (i) the effect of starting basic education a year earlier; (ii) the effect of extending basic education by an extra year; and (iii) the effect of taking an additional year of higher education. The meta-analysis reported that effects were smaller at higher stages of education: compared with estimated gains of 5.23 IQ points for children having started school a year earlier, and 2.06 IQ points for an additional year of basic education, the estimated gain from an additional year of higher education was 1.20 IQ points. It has been argued that even small effects of education are ‘potentially of great consequence’ [1, p. 1368]. But can we trust that there is even a small effect of higher education, rather than none at all? The reason for our concern is a serious weakness in the research design.

Effects of basic education on intelligence have been studied using strong research designs in the form of natural experiments. Studies of the effect of starting basic education a year earlier capitalize on the school-age cut-off, that is, the fact that the year in which a child enters school depends on their date of birth. Children that differ in age by a few months will differ in the time they have been in school either by a full year or not at all. By comparing intelligence between these two cases, the effect on children’s intelligence of having been an extra year in school can be estimated. Studies of the effect of extending basic education by an extra year instead capitalize on policy changes in which the minimum compulsory level of schooling is increased. Such policy changes lengthen the education of individuals who would otherwise have attended school at the pre-existing minimum compulsory level. Through comparison of the intelligence in pre-reform and post-reform cohorts, the effect on intelligence of extending basic education by an extra year can be estimated.

Because higher education is not mandatory, there is no corresponding population-wide natural experiment. Instead, studies of the effect of higher education on intelligence rely on longitudinal observations. Unfortunately, it is difficult to obtain trustworthy estimates from observational data of the effect of higher education on intelligence. The fundamental problem is to disentangle this effect from the reverse effect of more intelligent young people being more likely to progress to higher education levels, that is, a selection effect [2].

**Figure 1.**
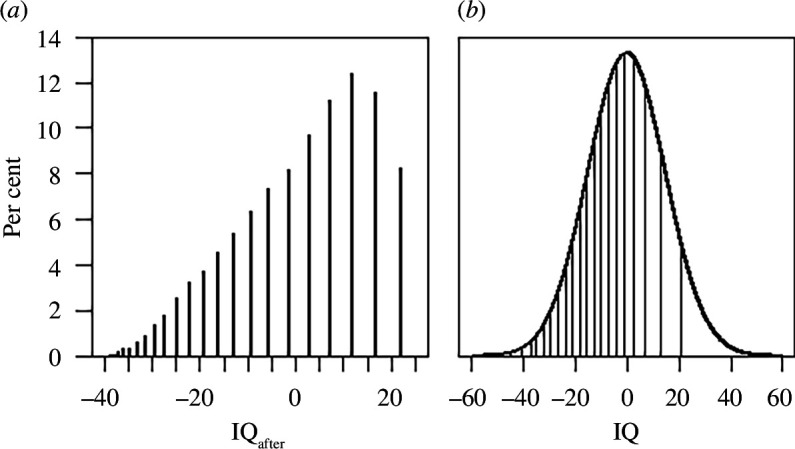
(*a*) The discrete distribution of the measure of intelligence after higher education used by Ritchie and Tucker-Drob [1]. (*b*) Segments of the normal distribution to which the different unique values of the discrete measure correspond.

The standard method for dealing with the selection effect is to include a childhood measure of intelligence as a covariate in a linear regression of intelligence on years of higher education. This is the method used in all eight studies of the effects of higher education on intelligence included in the meta-analysis by Ritchie and Tucker-Drob [1]. Unfortunately, the covariate method (cov) is likely to yield a considerable overestimate of the effect of higher education on intelligence. The reason is that the measure of intelligence taken in childhood has limited reliability as a measure of intelligence at the start of higher education. The inclusion of childhood intelligence as a covariate will therefore only partially remove the selection effect. The implication is that part of the selection effect will incorrectly be counted as an education effect.

It has been known for half a century that the cov method to control for pre-treatment differences will produce spurious findings when measures are unreliable [3,4]. However, published research on the effects of higher education on intelligence rarely acknowledges the inherent bias and the possibility that findings are spurious. None of the eight studies included in the aforementioned meta-analysis attempted to account for the limited reliability of intelligence measures. This is a weakness of the literature because there is a standard method for accounting for measurement errors, often called errors-in-variables (eiv) regression [5]. This method can be applied to pre- and post-test designs like the ones discussed here [6]. As estimates of the reliability of intelligence tests exist, it would be a step forward to apply eiv regression in studies of the effect of higher education on intelligence.

Accounting for limited reliability may, however, be insufficient. Tests used to measure intelligence may have other problems that cause further misestimation of the effect of higher education. Here, we shall focus on one such problem that is present in the study by Ritchie and Tucker-Drob [1]. They used data from a large cohort study that included a large intelligence test at age 10 and a more limited numeracy assessment at age 34. The latter test was used as a measure of adult intelligence. Although numeracy and intelligence are strongly correlated, they are not equivalent. It is possible that the effect of higher education on numeracy differs from the effect on intelligence, but we cannot examine this possibility without an independent measure of intelligence. Our working assumption will be that numeracy and intelligence are in fact equivalent. Even under this assumption, estimates of the effect of higher education will be biased when scores on the numeracy test are used to measure adult intelligence. The reason is the limited ability of the numeracy test to discriminate between different levels of intelligence.

Specifically, while IQ scores are assumed to be normally distributed, the distribution of scores on the numeracy test is discrete (only 23 levels) and left-skewed (see figure 1*a*). To see how this property of the test biases results, consider children at the high end of the intelligence distribution. Because of the selection effect, these children will be over-represented among those who receive a long higher education. Any positive effect of higher education on their intelligence will go undetected because they cannot get more than full points at the numeracy test. A general method to account for ceiling effects in regression analyses is to use censored (also known as Tobit) regression [7]. However, for the present problem, it is not sufficient to use censored regression because the problem is the discretization as such and not just the ceiling effect. For example, not only the highest but also the second highest level of numeracy scores corresponds to a wide range of IQ scores (see figure 1*b*). To deal with the discretization problem, we may capitalize on the fact that, by the definition of IQ, we know that a proper IQ test given in the adult population would have produced a normal distribution with the same mean and standard deviation as the IQ scores in childhood. Thus, our dependent variable is a discretization of a known normal distribution, and a regression method for this case is required.

This article aims to reanalyse a dataset previously used to estimate the longitudinal effect of higher education on intelligence. We use the cov method, which is predominant in the field, as well as the eiv method, which accounts for limited reliability. Further, we develop an iterated simulations model (ism) which accounts for discretization of IQ test data. We compare the performance of these three models on simulated data as well as on data used by Ritchie and Tucker-Drob [1].

### 1.1. A model of observed and latent variables

**Figure 2.**
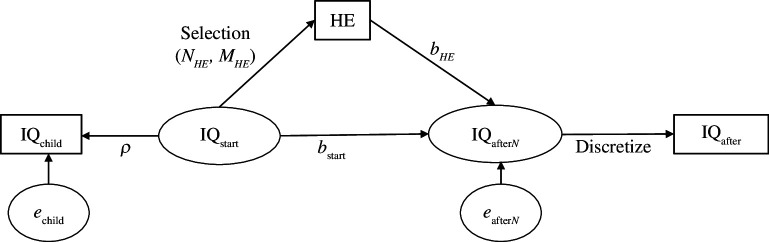
The model. Variables in rectangles are observed.

Figure 2 illustrates our model involving three observed and two latent variables. IQ_child_ is IQ measured in childhood, HE is the length of higher education (years) and IQ_after_ is adult IQ scores obtained at some point after people would have completed their higher education using a test that results in a discrete distribution of scores. Latent variables are IQ_start_, true IQ at the start of higher education, and IQ_after*N*_, the normally distributed adult IQ score that would have been obtained had the adult test been a proper IQ test. All IQ variables are assumed to be standardized to have a mean of 0 and s.d. of 15, so that var(IQ_child_) = var(IQ_start_) = var(IQ_after*N*_) = var(IQ_after_) = 225. The variable for higher education (HE) is measured in years but centred on the mean.

We write (*i*) after a variable when referring to the value for a specific individual *i*. The relationship between IQ_child_ and IQ_start_ depends on a reliability parameter *ρ*,


(1.1) $$IQ_{child}=\rho \;IQ_{start}\left(i\right)+e_{child}\left(i\right),$$

where the error terms *e*_child_(*i*) are assumed independently drawn from a normal distribution with mean 0 and variance equal to var(IQ_child_) – var(*ρ*IQ_start_).

In the dataset we analyse, HE can only take the values 0, 2, 6 and 10. The selection effect is described by a pair of parameter vectors *N*_HE_ *=* (*N*_0_, *N*_2_, *N*_6_, *N*_10_) and *M*_HE_ *=* (*M*_0_, *M*_2_, *M*_6_, *M*_10_), where *N_l_* is the number of individuals with HE = *l* and *M_l_* is the expected value of IQ_start_ among those individuals, that is,


(1.2) $$M_{l}=E\;\left[IQ_{start}\left(i\right)|\;HE\left(i\right)=l\right].$$

The effect of higher education on adult IQ is assumed to be *b*_HE_ IQ points per year of higher education. Specifically, properly measured adult IQ is assumed to be described by the following linear model:


(1.3) $$IQ_{afterN}\;\left(i\right)=b_{HE}HE\;\left(i\right)+b_{start}IQ_{start}\;\left(i\right)+e_{afterN}\;\left(i\right),$$

where the parameter *b*_start_ describes the contribution of IQ at the start of higher education to IQ after higher education and the error terms *e*_after*N*_(*i*) are assumed independently drawn from a normal distribution with mean 0 and variance equal to var(IQ_after*N*_) – var(*b*_HE_HE*+b*_start_IQ_start_).

The observed measure of adult IQ is a discretized version of IQ_after*N*_ in which whole segments of the normal distribution are mapped to the same value (figure 1*b*). We write this relationship as


(1.4) $$IQ_{after}=Discretize\;\left(IQ_{afterN}\right).$$

We may express IQ_after_(*i*) using the same form as equation (1.2),


(1.5) $$IQ_{after}\left(i\right)=b_{HE}HE\left(i\right)+b_{start}IQ_{start}\left(i\right)+e_{after}\left(i\right).$$

However, the error terms *e*_after_(*i*) are not independently drawn. Instead, *e*_after_ is determined by IQ_start_, HE and *e*_after*N*_ as follows:


(1.6) $$e_{after}=Discretize\;\left(b_{HE}HE+b_{start}{IQ}_{start}+e_{afterN}\right)–\left(b_{HE}HE+b_{start}IQ_{start}\right).$$

### 1.2. Estimating *b*_HE_ from observed variables

The situation we consider is that we have data on the observed variables and assume that they have been generated under the model described above. The goal is to estimate *b*_HE_ and *b*_start_ by finding the values of these parameters under which the model is expected to produce the observed covariances between the observed variables.

#### 1.2.1. The covariate method

In the special case of IQ_child_ = IQ_start_ and IQ_after_ = IQ_after*N*_, the estimation goal would be achieved by linear regression of IQ_after_ on HE with IQ_start_ as a covariate. As explained in any textbook on linear regression [8], the resulting estimate for *b*_HE_ and *b*_start_ are


(1.7) $$b_{HE\left(cov\right)}=\frac{var\left(IQ_{child}\right)cov\left(IQ_{after},HE\right)-cov\left(IQ_{child},HE\right)cov\left(IQ_{after},IQ_{start}\right)}{var\left(IQ_{child}\right)var\left(HE\right)-cov(IQ_{child,}HE{)}^{2}}$$

and


(1.8) $$b_{start\left(cov\right)}=\frac{var\left(HE\right)cov\left(IQ_{after},IQ_{child}\right)-cov\left(IQ_{child},HE\right)cov\left(IQ_{after},HE\right)}{var\left(IQ_{child}\right)var\left(HE\right)-cov{\left(IQ_{child},HE\right)}^{2}}.$$

The crux is that the equalities IQ_child_ = IQ_start_ and IQ_after_ = IQ_after*N*_ do not hold in our model, so equations (1.7) and (1.8) are expected to yield biased estimates.

#### 1.2.2. The error-in-variables method

Now consider the special case of IQ_after_ = IQ_after*N*_ and the known reliability parameter *ρ*. When the value of *ρ* is known, the error in IQ_child_ can be corrected using eiv regression. The resulting estimates for *b*_HE_ and *b*_start_ are


(1.9) $$b_{HE\left(eiv\right)}=\frac{\rho^{2}var\left(IQ_{child}\right)cov\left(IQ_{after},HE\right)-cov\left(IQ_{child},HE\right)cov\left(IQ_{after},IQ_{child}\right)}{\rho^{2}var\left(IQ_{child}\right)var\left(HE\right)-cov(IQ_{child,}HE{)}^{2}}$$

and


(1.10) $$b_{start\left(eiv\right)}=\rho \frac{var\left(HE\right)cov\left(IQ_{after},IQ_{child}\right)-cov\left(IQ_{child},HE\right)cov\left(IQ_{after},HE\right)}{\rho^{2}var\left(IQ_{child}\right)var\left(HE\right)-cov(IQ_{child,}HE{)}^{2}}.$$

These formulae have been used to adjust for the limited reliability of pretest scores in other contexts [6,9]. Note that if we set *ρ* = 1 (perfect reliability) in these equations, we recover equations (1.7) and (1.8). Thus, eiv regression is a generalization of linear regression. The crux is that the equality IQ_after_ = IQ_after*N*_ does not hold in our model. As explained by texts on eiv regression [5], this method is derived under the assumption that errors in the dependent variable are independent of the independent variables. In our model, this assumption does not hold, because *e*_after_ depends on HE and IQ_start_ as shown in equation (1.6). Hence, equations (1.9) and (1.10) are expected to yield biased estimates.

#### 1.2.3. The iterated simulations method

We shall now present a method to obtain unbiased estimates using an ism method. In appendix A, we derive the following equations that correct the eiv estimates for dependencies between the error terms *e*_after_ and the independent variables HE and IQ_start_


(1.11) $$b_{HE\left(ism\right)}=\frac{\rho^{2}var\left(IQ_{child}\right)\left(cov\left(IQ_{after},HE\right)-cov\left(e_{after},HE\right)\right)-cov\left(IQ_{child},HE\right)\left(cov\left(IQ_{after},IQ_{child}\right)-cov\left(e_{after},IQ_{child}\right)\right)}{\rho^{2}var\left(IQ_{child}\right)var\left(HE\right)-cov(IQ_{child,}HE{)}^{2}},$$

(1.12) $$b_{start\left(ism\right)}=\rho \frac{var\left(HE\right)\left(cov\left(IQ_{after},IQ_{child}\right)-cov\left(e_{after},IQ_{child}\right)\right)-cov\left(HE,IQ_{child}\right)\left(cov\left(IQ_{after},HE\right)-cov\left(e_{after},HE\right)\right)}{\rho^{2}var\left(IQ_{child}\right)var\left(HE\right)-cov(IQ_{child,}HE{)}^{2}}.$$

**Figure 3.**
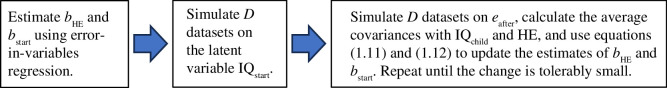
Summary of the iterated simulations method to estimate the effect of higher education on intelligence.

Note that if we assume cov(*e*_after_, HE) = 0 and cov(*e*_after_, IQ_child_) = 0, we recover equations (1.9) and (1.10). Thus, equations (1.11) and (1.12) generalize the eiv method. As the error terms *e*_after_ are not observed, we cannot directly obtain estimates of *b*_HE_ and *b*_start_ from these equations. However, given initial estimates of *b*_HE_ and *b*_start_, we can simulate data on the error terms and use the equations to update the estimates. This procedure can be iterated until the values have converged. This method of iterated simulations is summarized in figure 3.

The first step is to make initial estimates of *b_HE_* and *b*_start_ using eiv regression (equations (1.9) and (1.10)).

The second step is to simulate data on the latent variable IQ_start_ to fit both the model and the observed data on HE and IQ_child_. As shown in appendix A, this is achieved by the generating equation,


(1.13) $$IQ_{start}^{sim}\left(i\right)=\beta M_{child}\left(i\right)+\left(\rho^{-1}–\beta \right)IQ_{child}\left(i\right)+e_{start}\left(i\right),$$

where the supplementary parameter *β* and supplementary variable *M*_child_ are calculated from observed data as described in appendix A. The error terms *e*_start_(*i*) are drawn independently from a normal distribution with mean of 0 and variance equal to 225 – var(*β M*_child_ + (*ρ* ^−1^ – *β*) IQ_child_).

Due to the random draws, every simulated dataset yields somewhat different results. Below, errors can be reduced by the simulation of several (*D*) datasets. Our demonstration below suggests that *D* = 25 datasets are enough.

Given guesses of *b*_HE_ and *b*_start_, we use the data on HE and the *D* simulated datasets on IQ_start_ in equation (1.6) to obtain *D* simulated datasets of *e*_after_. The average values of the covariances cov(*e*_after_, IQ_child_) and cov(*e*_after_, HE) across the *D* datasets are used in equations (1.11) and (1.12) to yield updated values of *b*_HE_ and *b*_start_. This step is iterated until the change in values is smaller than a preset tolerance.

This method is an example of fixed-point iteration. When it converges, it finds a fixed point, which in the case at hand means a pair of values *b*_HE_ and *b*_start_ such that data generated with these parameter values satisfy equations (1.11) and (1.12). Fixed-point iteration methods do not necessarily converge. However, our analyses demonstrate convergence in this case.

## 2. Analysis of simulated data

We use simulated data to demonstrate the relative accuracy of the three methods across various parameter values, selection effects and discretizations. This section is divided into two parts. In the first part, we simulate data in a toy model where the sample size is 1000, the length of higher education only takes two different values (0 or 1 year), and the discrete distribution of the adult intelligence measure is either uniform or triangular. Data are simulated under set true values of the parameters *ρ*, *b*_HE_ and *b*_start_. We then apply the cov method, the eiv method and the ism method to the simulated data to obtain estimates of *b*_HE_, which we compare with the true value of this parameter.

In the second part, we simulate data that matches the real data as closely as possible. The aims of this part are to demonstrate what results to expect in the real data if the model is correct and what results to expect if the true effect of higher education on intelligence is nonlinear. The simulation of data and subsequent analysis were implemented in R 4.0.2 [10]. The R scripts are available as electronic supplementary material.

### 2.1. Analysis of simulated data in a toy model

We simulate data on a sample of *n* = 6000 individuals. Data on IQ_start_ are drawn from a normal distribution with a mean of 0 and s.d. of 15. Data on IQ_child_ are simulated using equation (1.1) with the value of the reliability parameter *ρ* set to either 0.84 or 1 (i.e. perfect reliability so that IQ_child_ = IQ_start_). The objective is to demonstrate that imperfect reliability is corrected by the ism method and the eiv method, whereas it leads to bias in the cov method.

Data on HE are simulated using either of two selection schemes, *p*-selection or *p* ^2^-selection, defined as follows. With *p*(*i*) representing the proportion of individuals who have a lower intelligence than individual *i* at the start of higher education, the *p*-selection scheme is to set HE(*i*) to 1 with probability *p*(*i*) and 0 otherwise. Simulations show that *p*-selection is characterized by parameter values *N*_0_ = 3000, *N*_1_ = 3000, *M*_0_ *=* –8.46 and *M*_1_ = 8.46 (see equation (1.2)). The alternative selection scheme is *p* ^2^-selection, in which HE(*i*) is set to 1 with probability (*p*(*i*))^2^ and 0 otherwise. Simulations show that *p* ^2^ selection is characterized by *N*_0_ = 4000, *N*_1_ = 2000, *M*_0_ *=* –6.35 and *M*_1_ = 8.46. The purpose of including more than one selection scheme is to demonstrate that the biases of the cov and eiv methods are moderated by the selection effect, whereas the ism method always yields unbiased estimates under our model.

Data on IQ_after*N*_ are simulated using equation (1.3) with the value of parameter *b*_start_ fixed to 0.6 but with the effect of higher education varying between simulations to have either value *b*_HE_ = 4 or *b*_HE_ = 8. The objective is to demonstrate that all estimation methods produce higher estimates when the true value is higher, but the bias in the eiv and cov methods may vary with the true value.

Data on IQ_after_ are simulated in either of three ways: by setting IQ_after_ = IQ_after*N*_ (no discretization) or by discretizing IQ_after*N*_ to four equidistant levels with 250 individuals at each level (uniform discretization) or by discretizing IQ_after*N*_ to four equidistant levels with 100 individuals at the lowest level, 200 at the second level, 300 at the third level and 400 at the highest level (triangular discretization). The discrete distributions are standardized to have mean of 0 and s.d. of 15. The objective is to demonstrate that while the ism method corrects for any discretization, the biases of the cov and eiv methods depend on how data discretized.

For each combination of parameter values, selection scheme and discretization scheme, we simulate 1000 datasets. In each dataset we estimate the value of *b*_HE_ using three different methods: the cov method (equation (1.7)), the eiv method using the true reliability *ρ* (equation (1.9)) and the ism method using the true reliability *ρ* and *D* = 25 simulated datasets to estimate the error covariances used in equations (1.11) and (1.12), stopping iterations when the change between subsequent estimates is less than 0.01 (which usually occurred after four or five iterations).

**Figure 4.**
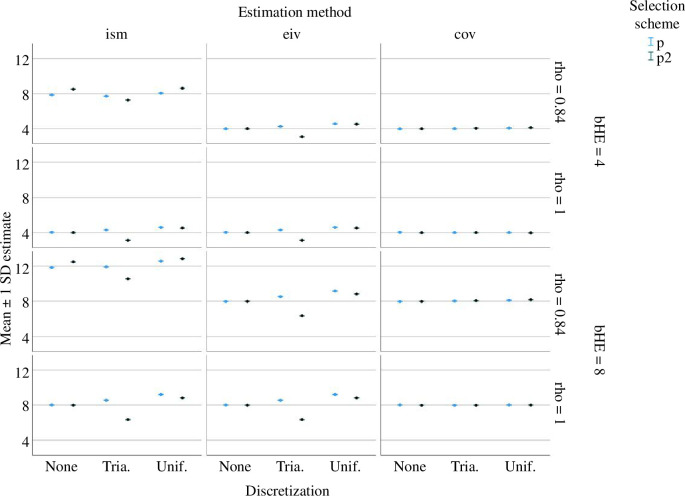
Mean values (±1 s.d.) of estimates of *b*_HE_ when applying the iterated simulations method (ism), the errors-in-variables method (eiv) and the covariate method (cov) to 1000 simulated datasets for each combination of a true value of *b*_HE_ (4 or 8), a reliability level *ρ* (1 or 0.84), a selection scheme (*p* or *p* ^2^) and a discretization (none, uniform or triangular).

Figure 4 summarizes the results. Note that the ism method yields unbiased estimates of *b*_HE_ for any value of reliability, any selection scheme and any discretization scheme. By contrast, estimates obtained using the eiv method are biased whenever IQ_after_ is discretized. The bias in these estimates may be either positive or negative depending on the specification of the selection and discretization schemes, and the amount of bias depends on the reliability value. The complex pattern of the bias in the eiv method demonstrates that correction requires a method that simultaneously takes into account the specific discretization scheme, the specific selection effect and the specific reliability level. This is what the iterated simulation method does. Figure 4 also shows that the cov method produces severely biased estimates when the reliability is low, whereas it is equivalent to the eiv method when reliability is perfect.

### 2.2. Analysis of simulated data matching the real data

Next, we simulate data matching the real data as closely as possible (the real data are described in detail below). For a set of *n* = 6766 individuals, we use real data on the observed variables IQ_child_ and HE (*N*_0_ = 3877, *N*_2_ = 655, *N*_6_ = 1808 and *N*_10_ = 426). We simulate data on IQ_start_ using equation (1.13) with the reliability parameter *ρ* set to 0.84. The expected mean values of IQ_start_ at different lengths of higher education are *M*_0_ *=* –6.2, *M*_2_ *=* 2.8, *M*_6_ *=* 8.1 and *M*_10_ *=* 18.0.

Data on IQ_after*N*_ are simulated using equation (1.3) with the value of parameters *b*_start_ and *b*_HE_ set to 0.6 and 0.45, respectively. In an alternative set of simulations of IQ_after*N*_, we replace the linear effect of 0.45 IQ points per year of higher education with a nonlinear effect: 2.4 points after the first 2 years, 3.1 points after 6 years and 3.8 points after 10 years (i.e. the effect per year is much higher in the first 2 years than in the subsequent years). This specific nonlinear effect was chosen because it yields the same effect estimate as the linear model. Data on IQ_after_ are simulated through the discretization of IQ_after*N*_ to the observed distribution of IQ_after_ in the real data (see figure 1). The objective is to examine how well the ism method, which assumes a linear effect, fits the data when the true effect is nonlinear.

For each specification of the effect of higher education, we simulate 1000 datasets and estimate the value of *b*_HE_ using the same three methods as above. The iterated simulation method correctly yields a mean estimate, 0.45, whereas the cov method produces an almost twice as high mean estimate, 0.89, while the eiv method yields a too low mean estimate, 0.39. The same estimates were obtained when the true effect was nonlinear.

For each length of higher education, we also compared the mean adult IQ score between the simulated ‘true’ data and the simulated data in the final iteration of the ism method. When the true effect is linear, there is little difference. When the true effect is nonlinear, however, true adult scores for individuals with 2 years of higher education are on average more than 1 IQ point higher than the corresponding scores estimated by the ism method (table 1).

**Table 1.** Differences between ‘true’ simulated data and data in the final iteration of the iterated simulations method with respect to mean adult IQ scores for various lengths of higher education.

true effect	0 year	2 years	6 years	10 years
linear: *b*_HE_ = 0.45 points/year	0.1 (0.1)	−0.1 (0.5)	−0.1 (0.2)	−0.2 (0.4)
nonlinear: 2.6 points after 2 years, 3.2 points after 6 years and 3.8 points after 10 years	−0.1 (0.1)	1.2 (0.4)	0.0 (0.3)	−0.9 (0.6)

*Notes:* Entries are mean differences with s.d. within parentheses.

## 3. Reanalysis of data from the 1970 British Cohort Study

We next reanalyse data from the 1970 British Cohort Study [11,12]. This is a longitudinal dataset that has previously been used to estimate the effect of education on intelligence [1].

### 3.1. Data

#### 3.1.1. Participants

The original sample (achieved sample size *n* = 16 571) consisted of all individuals born in England, Northern Ireland, Scotland and Wales during a single week in April 1970 (those born in Northern Ireland were dropped from subsequent sweeps). Of the original participants, 14 874 and 9656 participated in the second and the sixth sweeps at ages 10 and 34, respectively [13]. In the present analyses, we use data from 6766 individuals (47.2% male) who satisfied the following inclusion criteria: (i) not twins; (ii) data were available on length of education; and (iii) non-zero scores were available on all measurements of intelligence.^1^

#### 3.1.2. Length of education

At age 34, participants were asked about their highest obtained educational qualifications. Adapting the coding scheme used by Ritchie and Tucker-Drob [1], we converted these qualifications to *years of higher education* as follows: (i) no qualifications, CSE, GCSE or O-level = 0 year (*N*_0_ = 3877); (ii) A-level, SSCE or AS-level = 2 years (*N*_2_ = 655); (iii) degree, diploma of higher education, other teaching qualifications or nursing qualifications = 6 years (*N*_6_ = 1808); and (iv) higher degree or PGCE = 10 years (*N*_10_ = 426). This is the variable *HE* (*M* = 2.43 years, s.d. = 3.23 years).

#### 3.1.3. Intelligence measure in childhood

At age 10, participants completed four subscales of the British Ability Scales: (i) *Word definitions*—participants were asked to define words from simple (e.g. ‘travel’, 82.6% correct) to difficult (‘hirsute’, 0.1% correct), 37 items; (ii) *Recall of digits*—participant were asked to remember two (‘44’, 99.9% correct) to eight (‘25 837 461’, 10.6% correct) digit numbers, 34 items; (iii) *Similarities*—the interviewer presented three words in a given category and the participant was required to give a fourth example in the same category and to name the category (e.g. ‘red, blue, brown’, 99.3% correct example and 99.1% correct name; ‘democracy, justice, equality’, 1.1% correct example and 0.6% correct name), 21 items; and (iv) *Matrices*—participants were asked to complete a pattern of figures by drawing the appropriate shape in the empty bottom-right square in a 2 × 2 or a 3 × 3 matrix, correct responses varied between 4.8% and 99.5%, 28 items. After standardization of the scores on each of the four tests, we calculated the average for each participant. We obtain IQ_child_ by standardizing this measure to an IQ metric centred on the mean (i.e. *M* = 0, s.d. = 15).

#### 3.1.4. Intelligence measure after higher education

At age 34, participants completed a numeracy assessment with 17 multiple-choice and 6 open-response numeracy questions. The questions measured the participants’ understanding of numbers, symbols, diagrams, charts and mathematical information. Following Ritchie and Tucker-Drob [1], we obtain IQ_after_ by standardizing the squared score on this test to an IQ metric centred on the mean. This measure has the discrete distribution shown in figure 1*a*.

#### 3.1.5. Estimation of the reliability parameter *ρ*

To estimate the reliability of IQ_child_ as a measure of IQ_start_, we use a summary of a large number of prior studies of the test–retest reliability of intelligence tests similar to the British Ability Scales ([14], table 4.) At ages 9 and 12, the estimated 6 years test–retest reliabilities are 0.81 and 0.84, respectively. By interpolating between these numbers, we estimate the test–retest reliability of tests at age 10 and 6 years later to be 0.82. However, the hypothetical test at age 16 would have some small measurement errors with respect to IQ_start_, which refers to *true* intelligence at age 16. In the same table, we find that the estimated 3-month test–retest reliability at age 15 is 0.94. The test–retest reliability on the same day would be higher, say 0.95. The correlation between the hypothetical test score at age 16 and true intelligence at age 16 may then be estimated to be 0.95^1/2^. The correlation of the test score at age 10 and true intelligence at age 16 is, therefore, estimated to be 0.82/0.95^1/2^ = 0.84. To capture the uncertainty, our working estimate will be *ρ* = 0.84 ± 0.02.

### 3.2. Analysis

Similar to the above analysis of simulated data, analysis of the real data was implemented in R 4.0.2 [10]. Estimates were obtained using the cov, eiv and ism methods applied to 1000 resamples of the real data from which mean estimates with 95% bootstrapped confidence intervals (CIs) were calculated. The R script is available at [15].

### 3.3. Results

Table 2 presents the estimates of *b*_HE_ and *b*_start_ obtained using the three methods. Our focus is on estimates of *b*_HE_, the effect of higher education on IQ. The cov method yields a *b*_HE_ estimate of 0.87 IQ points per year of higher education.^2^ This estimate is inflated due to the imperfect reliability of the measure of IQ before higher education. From prior data on the reliability of IQ tests, a plausible estimate of the reliability parameter is 0.84. Assuming this value, the eiv method yields a corrected estimate of 0.36. However, that estimate is biased downward due to the incorrect distribution of the measure of IQ after higher education. The corrected estimate obtained using the ism method is a bit higher, 0.42, but still less than half the estimate obtained using the cov method. Even taking sampling error and uncertainty in the reliability assumption into account, all plausible estimates are considerably below the estimate produced by the cov method.

**Table 2.** Results of three methods of estimating *b*_HE_ (the effect of higher education on IQ) and *b*_start_ (the contribution of IQ at the start of higher education to adult IQ) in real data.

assumed	covariate method		error-in-variables		iterated simulations	
value of *ρ*	*b*_HE_	*b*_start_	*b*_HE_	*b*_start_	*b*_HE_	*b*_start_
0.82	0.86 [0.76, 0.97]	0.47 [0.44, 0.50]	0.27 [0.11, 0.42]	0.64 [0.60, 0.68]	0.32 [0.16, 0.48]	0.65 [0.61, 0.69]
0.84	0.86 [0.76, 0.97]	0.47 [0.44, 0.50]	0.36 [0.22, 0.50]	0.62 [0.57, 0.66]	0.42 [0.27, 0.57]	0.62 [0.59, 0.66]
0.86	0.86 [0.76, 0.97]	0.47 [0.44, 0.50]	0.44 [0.31, 0.58]	0.59 [0.55, 0.63]	0.51 [0.37, 0.65]	0.60 [0.56, 0.64]

*Notes*: Estimates with 95% CIs based on 1000 bootstrap samples.

To assess how well the model fits the data, we compared mean IQ scores after different lengths of higher education in real data with the corresponding scores obtained in the simulated data in the final iteration of the ism method (table 3). Note that scores in the group with 2 years of higher education were more than 1 IQ point higher in the real data than in the simulated data. As we saw in the simulation study, this pattern is expected if the true effect of higher education is not linear but higher in the first 2 years and much lower thereafter.

**Table 3.** Differences between real and simulated data with respect to mean adult IQ scores for various lengths of higher education.

length of higher education	difference between real and simulated data in mean adult IQ		
	*ρ* = 0.82	*ρ* = 0.84	*ρ* = 0.86
0 year	−0.18 [−0.32, −0.03]	−0.21 [−0.34, −0.08]	−0.24 [−0.36, −0.10]
2 years	1.06 [0.12, 2.00]	1.31 [0.37, 2.24]	1.52 [0.59, 2.44]
6 years	0.06 [−0.28, 0.39]	0.06 [−0.27, 0.39]	0.04 [−0.27, 0.36]
10 years	−0.27 [−1.15, 0.53]	−0.33 [−1.19, 0.47]	−0.39 [−1.22, 0.39]

*Notes*: Entries are the mean value in real data minus the mean value in simulated data with 95% CIs based on 1000 bootstrap samples.

## 4. Discussion

In this article, we have brought attention to challenges associated with assessing the impact of higher education on intelligence. In the absence of natural experiments, researchers’ best option is to analyse data from longitudinal cohort studies. The main problem for such analyses is to distinguish the effect of higher education on intelligence from the selection effect, that is, the phenomenon that more intelligent people tend to progress further in the education system. Unless the selection effect is fully accounted for in the analysis, estimates of the effect of education on intelligence will be biased upward. To remove the selection effect is challenging, because the intelligence level on which the selection effect acts is unobservable, and so is the intelligence level achieved after higher education. Researchers, therefore, resort to intelligence measures taken sometime in childhood and adulthood, but these measures have limited test–retest reliability, especially across longer time spans. Adding to the complexity of our reanalysed dataset from the 1970 British Cohort Study was that the adult intelligence measure had an incorrect, discrete distribution so that measurement errors are non-classical.

Many studies have ignored these complications when using an analytical strategy in which adult intelligence is regressed on the length of higher education with childhood intelligence included as a covariate [1,16–19]. Estimates from these studies of a positive effect of higher education on intelligence, typically around 1 IQ point per year of education, are therefore likely to have been exaggerated. In this article, we have addressed the analytical problem more thoroughly.

The first, and most important, improvement of the analytical strategy is to take the measurement errors in childhood intelligence measures into account. This requires two steps: the first step is to use known test–retest reliabilities of intelligence tests to estimate how reliably the childhood intelligence score represents the intelligence at the start of higher education, while the second step is to replace the cov method with eiv regression using the estimated reliability. In our study, this reduced the estimate of the effect of higher education on intelligence by more than 50% to less than 0.4 IQ points per year of higher education.

This estimate may still be biased in the case where the adult intelligence measure does not produce a properly normally distributed score. For this case, we developed an ism method that corrects for incorrectly distributed adult intelligence scores. Analysis of simulated data showed that the ism method produces unbiased effect estimates. Applied to the data from the 1970 British Cohort Study, the ism method estimated the effect to around 0.4 IQ points per year of higher education, slightly higher than the estimate obtained from eiv regression.

Following Ritchie and Tucker-Drob [1], we used a numeracy assessment in the 1970 British Cohort Study as a measure of adult intelligence. The validity of our effect estimates rests on the questionable assumption that the only problem with using this numeracy assessment as an intelligence measure is that its scores are not correctly distributed. As numeracy is not equivalent to intelligence, results might have been different had a proper intelligence test been available. However, from the meta-analysis of Ritchie and Tucker-Drob [1], we know that the estimate of the effect of higher education obtained in this dataset (using the cov method) is close to the meta-analytic average. Thus, the numeracy assessment appears to produce similar results to the tests of adult intelligence that were used in other studies. A direction for future research would be to reanalyse all the datasets included in the meta-analysis along the lines in this article, and to compare the results for studies using different types of tests of adult intelligence. Such an analysis should also include any more recent studies not included in the meta-analysis (e.g. [20]).

Another questionable assumption is that the effect of higher education on intelligence is linear, that is, every additional year of higher education produces the same increase in intelligence. If the true effect of higher education is not linear, the estimate from a linear model only represents a weighted average of the effect of different lengths of education. There is reason to believe that the effect of education is diminishing, because Ritchie and Tucker-Drob found much larger effects in studies of basic education than in studies of higher education and noted: ‘We might expect the marginal cognitive benefits of education to diminish with increasing educational duration, such that the education–intelligence function eventually reaches a plateau’ [1, p. 1367]. In line with a larger effect of the first 2 years of higher education and a smaller effect of subsequent education, we found the linear model to underestimate the intelligence in the group with 2 years of higher education.

There may be other kinds of model misspecification too. For one thing, the reliability of intelligence measurement may depend on the intelligence level. For another, there may be confounding factors, such as parents’ profession and education, that affect both the longitudinal development of intelligence and the likelihood of completing more years of higher education. Analysis of the impact of such additional confounders is beyond the scope of this article, but if they exist, accounting for them would probably reduce the effect estimate further.

In conclusion, we found that a longitudinal dataset used in a previous study to estimate the effect of higher education on intelligence had childhood intelligence measures of limited reliability and adult intelligence measures with dependent measurement errors. We developed an iterated simulations method to account for these limitations of the data. In a reanalysis of the data using this method, the estimated effect of higher education dropped to approximately half the size, indicating that prior estimates have been considerably exaggerated. Future meta-analytic estimations of the effect of higher education on intelligence should consider the reliability and any discretization of measures. We have here developed the means to do so.

## Data Availability

All data used in this study were from the 1970 British Cohort Study and are available from the UK Data Service [21]. The data collection is available to users registered with the UK Data Service. Electronic supplementary material is available online [15].

## Footnotes

The study by Ritchie and Tucker-Drob [1] included fewer participants in the analysis (*n* = 5296). Their inclusion criteria were not reported.

The study by Ritchie and Tucker-Drob [1] on this dataset included sex as a covariate as well, which led to a marginally higher estimate of 0.92 for the effect of education.

## Appendix A.

In the below equations, ‘plim’ in front of equalities refers to the probability limit, that is, the left-hand and right-hand sides of equalities converge in probability as the sample size tends to infinity.

### A.1. Derivation of equations (1.9) and (1.10)

Under our model we can derive a version of the so-called normal equations [8]. When variables are centred so that their expected values are 0, variances and covariances satisfy the equations plim var(*X*) = *E*[*X* ^2^] and plim cov(*X*,*Y*) = *E*[*XY*]. Using these equations and the model equations, we can express covariances involving the unobserved variable IQ_start_ in terms of observed covariances as follows. By multiplying equation (1.1) with IQ_start_ and taking expected values, we obtain

(A 1)
$$plim\;cov\left(IQ_{start},IQ_{child}\right)=\rho \;var\left(IQ_{start}\right)=\rho \;var\left(IQ_{child}\right),$$

where the last equality follows from the standardization of IQ scores. By multiplying equation (1.1) with HE and taking expected values, we obtain

(A 2)
$$plim\;cov\left(IQ_{start},HE\right)=\rho^{-1}cov\left(IQ_{child},HE\right).$$

We can then express covariances involving the dependent variable IQ_after_ as follows. By multiplying equation (1.5) with HE*,* taking expected values, and applying equation (A 2), we obtain

(A 3)
$$plim\;cov\left(IQ_{after},HE\right)=b_{HE}var\left(HE\right)+b_{start}\rho^{-1}cov\left(IQ_{child},HE\right)+cov\left(e_{after},HE\right).$$

By multiplying equation (1.5) with IQ_child_ *,* taking expected values, and applying equation (A 1), we obtain

(A 4)
$$plim\;cov\left(IQ_{after},IQ_{child}\right)=b_{HE}cov\left(HE,IQ_{child}\right)+b_{start}\;\rho \;var\left(IQ_{child}\right)+cov\left(e_{after},IQ_{child}\right).$$

Equations (A 3) and (A 4) constitute a system of linear equations for the parameter values *b*_HE_ and *b*_start_, which can be solved by standard methods in linear algebra. Equations (1.11) and (1.12) describe the solution.

### A.2. Verification of equation (1.13) for simulating data on IQ_start_

Our method rests on the claim that under our model, data on IQ_start_ can be simulated to fit observed data using equation (1.13),

(A 5)
$$IQ_{start}^{sim}\left(i\right)=\beta M_{child}\left(i\right)+\left(\rho^{-1}-\beta \right)IQ_{child}\left(i\right)+e_{start}\left(i\right).$$

The supplementary variable *M*_child_ is calculated from observed data as the mean value of IQ_child_ across all individuals that have the same length of higher education:,

(A 6)
$$M_{child}\left(i\right)=E\left[IQ_{child}\left(j\right)\;|\;HE\left(j\right)=HE\left(i\right)\right].$$

The supplementary parameter *β* is then calculated as follows:

(A 7)
$$\beta =var\left(IQ_{child}\right)\left(\rho^{-1}-\rho \right)/\left(var\left(IQ_{child}\right)-cov\left(IQ_{child},M_{child}\right)\right).$$

We shall verify that the simulated variable IQ_start_^sim^ matches the unobserved variable IQ_start_ with respect to how they relate to IQ_child_ and HE.

First, we need to check that IQ_start_^sim^ and IQ_start_ have the same expected covariance with IQ_child_.

plim cov(IQ_start_^sim^ *,*IQ_child_) =

{use equation (1.13)} = *β* cov(IQ_child_, *M*_child_) +(*ρ* ^−1^ – *β*)var(IQ_child_)

{use equation (A 6)} = *ρ* var(IQ_child_)

{use equation (A 1)} = plim cov(IQ_start_ *,*IQ_child_).

Second, if we define *M_l_* ^sim^ as the mean value of IQ_start_^sim^ among individuals with HE = *l*, we need to check that the expected values of *M_l_* ^sim^ are equal to the expected values of *M_l_*.

plim *E*[*M_l_* ^sim^] =

{use equation (1.13)} = *E*[(*β M*_child_(*i*) + (*ρ* ^−1^ – *β*) IQ_child_(*i*)) | HE(*i*) = *l*]

{use equation (A 5)} = *E*[*ρ* ^−1^ IQ_child_(*i*) | HE(*i*) = *l*]

{use equation (1.1)} = *E*[IQ_start_(*i*) | HE(*i*) = *l*]

{use equation (1.2)} = *E*[*M_l_*]
